# Acute irradiation causes a long-term disturbance in the heterogeneity and gene expression profile of medullary thymic epithelial cells

**DOI:** 10.3389/fimmu.2023.1186154

**Published:** 2023-11-02

**Authors:** Kenta Horie, Kano Namiki, Kyouhei Kinoshita, Maki Miyauchi, Tatsuya Ishikawa, Mio Hayama, Yuya Maruyama, Naho Hagiwara, Takahisa Miyao, Shigeo Murata, Tetsuya J. Kobayashi, Nobuko Akiyama, Taishin Akiyama

**Affiliations:** ^1^ Laboratory for Immune Homeostasis, RIKEN Center of Integrative Medical Sciences, Yokohama, Japan; ^2^ Graduate School of Pharmaceutical Sciences, The University of Tokyo, Tokyo, Japan; ^3^ Immunobiology, Graduate School of Medical Life Science, Yokohama City University, Yokohama, Japan; ^4^ Graduate School of Engineering, The University of Tokyo, Tokyo, Japan; ^5^ YCI Laboratory for Immunological Transcriptomics, RIKEN Center for Integrative Medical Sciences, Kanagawa, Japan; ^6^ Institute of Industrial Science, The University of Tokyo, Tokyo, Japan

**Keywords:** thymus, thymic epithelial cell (TEC), AIRE, radiation, scRNA seq

## Abstract

The thymus has the ability to regenerate from acute injury caused by radiation, infection, and stressors. In addition to thymocytes, thymic epithelial cells in the medulla (mTECs), which are crucial for T cell self-tolerance by ectopically expressing and presenting thousands of tissue-specific antigens (TSAs), are damaged by these insults and recover thereafter. However, given recent discoveries on the high heterogeneity of mTECs, it remains to be determined whether the frequency and properties of mTEC subsets are restored during thymic recovery from radiation damage. Here we demonstrate that acute total body irradiation with a sublethal dose induces aftereffects on heterogeneity and gene expression of mTECs. Single-cell RNA-sequencing (scRNA-seq) analysis showed that irradiation reduces the frequency of mTECs expressing AIRE, which is a critical regulator of TSA expression, 15 days after irradiation. In contrast, transit-amplifying mTECs (TA-mTECs), which are progenitors of AIRE-expressing mTECs, and Ccl21a-expressing mTECs, were less affected. Interestingly, a detailed analysis of scRNA-seq data suggested that the proportion of a unique mTEC cluster expressing Ccl25 and a high level of TSAs was severely decreased by irradiation. In sum, we propose that the effects of acute irradiation disrupt the heterogeneity and properties of mTECs over an extended period, which potentially leads to an impairment of thymic T cell selection.

## Introduction

The thymus provides an environment for development of T cells. Thymic epithelial cells (TECs) are essential for development and selection of thymic T cells by presenting self-antigens and expressing some cytokines and signaling molecules (1). TECs are largely divided into two types based on localization: cortical TECs (cTECs) and medullary TECs (mTECs). cTECs are critical for proliferation and differentiation of early progenitors into CD4^+^CD8^+^ (DP) thymocytes and they also positively select DP thymocytes recognizing a complex of self-peptide and major histocompatibility complex (MHC) with moderate affinity, thereby leading to differentiation of DP thymocytes into CD4^+^CD8^–^ (CD4SP) or CD4^–^CD8^+^ (CD8SP) thymocytes. Subsequently, CD4SP and CD8SP thymocytes interact with mTECs, which ectopically express and present self-peptides derived from tissue-specific antigens (TSAs). CD4SP and CD8SP thymocytes interacting with TSA peptides with high affinity undergo negative selection, or in the case of CD4SP thymocytes, differentiate into regulatory T cells. Autoimmune regulator (AIRE) is a transcriptional regulator that controls TSA expression in mTECs (2). Because dysfunctional mutations of the *AIRE* gene cause onset of human autoimmune disease (3), it is most likely that TSA expression in mTECs is essential to suppress onset of autoimmune diseases (4).

Recent studies using single-cell RNA sequencing analysis (scRNA-seq) revealed a high degree of TEC heterogeneity in humans and mice (5–9). Bornstein et al. classified mTECs into four subsets (5). mTEC IV (thymic tuft cells) showing gene expression and chromatin structure similar to intestinal tuft cells have been reported (5, 10), in addition to mTEC I, mTEC II, and mTEC III subsets that corresponding primarily to Ccl21a^+^mTEC^lo^, Aire^+^mTEC, and post-Aire mTEC subsets, respectively. Michelson et al. investigated heterogeneity of post-Aire mTECs in more detail. Post-Aire mTECs are an assembly of various mimetic cells harboring signature genes expressed by extra-thymic cell types (11, 12). Several studies using scRNA-seq have suggested a unique mTEC cluster expressing a high level of cell cycle-related genes (6, 8, 9). A further study demonstrated that this cluster is equivalent to transit-amplifying cells (9), which are proliferative progenitors connecting stem cells and mature cells (13).

Cytoreductive therapies such as chemotherapy and irradiation are frequently used in cancer treatment. Irradiation causes acute involution of the thymus (14, 15). Thymic involution by irradiation is ascribed to severe and acute reduction of thymocytes, mainly of DPs. In addition to thymocytes, TECs are affected by irradiation. Notably, after this acute thymic involution, both thymocytes and TECs are homeostatically recovered (14–17). Some studies have provided mechanistic insights on the recovery of TECs and the resulting recovery of the thymic organ (14–16, 18). Several cytokines and growth factors were reportedly involved in the thymic regeneration. Dudakov et al. have shown that IL-22 from type 3 ILCs is required for TEC proliferation after irradiation (16). Wertheimer et al. showed that bone morphogentic protein 4 (BMP4) is expressed in thymic endothelial cells and promotes thymic recovery after irradiation (18). Mechanistically, BMP4 signaling in TECs causes increased expression of Foxn1, which is critical for TEC development and maintenance. In an earlier study, it was reported that keratinocyte growth factor (KGF) contributes to the thymic recovery from irradiation by enhancing the proliferation and function of thymic epithelial cells (19). Mechanistically, KGF signaling up-regulates the expression of several target genes necessary for TEC regeneration, such as BMP4. KGF activates the p53 pathway for transcriptional activation. Indeed, the transcription factor p53, which is activated by radiation-induced DNA damage, promotes the regeneration of medullary TECs (mTECs) after irradiation (20). In contrast to these positive regulators, TGF-β signaling in TECs inhibits thymic recovery in the early phase after irradiation (21).

Although the reduction of TEC frequency by irradiation was known, its effect on TEC heterogeneity and their individual phenotype was not fully characterized. In this study, we performed scRNA-seq analysis of TECs in murine thymus 15 days after sublethal total body irradiation. Data analysis showed that irradiation affects proportions of mTEC subsets. Pseudotime analysis of scRNA-seq data and BrdU decay kinetic analysis have suggested that the lifetime of Aire^+^mTECs was not affected, but its differentiation might be retarded by irradiation. Moreover, scRNA-seq suggested a selective decrease of an mTEC subset expressing *Ccl25* and a high level of TSAs in addition to a reduction in TSA expression level. Consequently, these data suggested that irradiation affects phenotypes and heterogeneity of mTECs, which may influence thymic selection.

## Results

### mTEC composition was disturbed after irradiation

**Figure 1:**
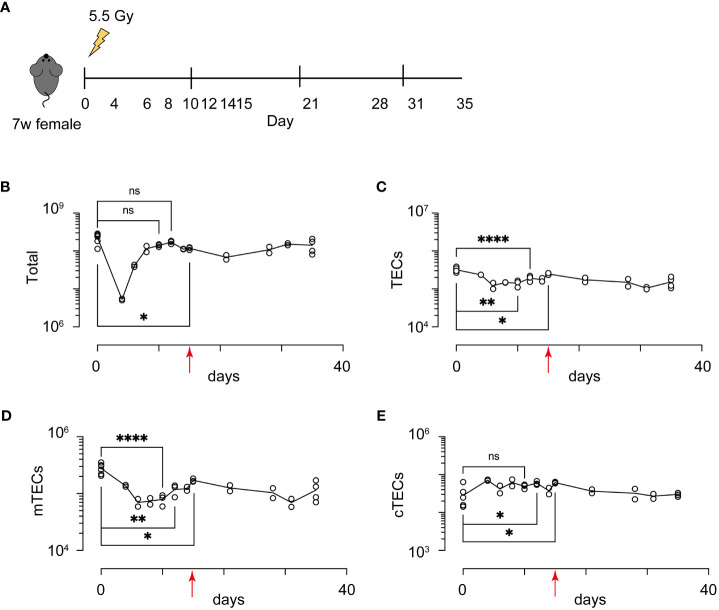
Acute sublethal irradiation caused a reduction and subsequent recovery in cellularity of thymic cells and thymic epithelial cells. **(A)** A schematic diagram of chase experiments of thymic cellularity after irradiation. Seven-week-old female mice with a C57BL/6 background were exposed to sublethal dose radiation. After the indicated days, total thymic cells were analyzed by flow cytometric analysis **(B-E)** Dynamics in cellularity were evaluated for total thymic cells **(B)**, TECs **(C)**, mTECs **(D)**, and cTECs **(E)** by flow cytometric analysis. Typical profiles of flow cytometric analysis were exhibited in **Supplementary Figure 1**. *p < 0.05; **p < 0.01; ****p < 0.0001; ns, p > 0.05; Student’s t-test. Red arrows indicate Day 15.

Because influences of radiation may be altered in different breeding environments, ages, genders, and genetic backgrounds, in addition to the radiation dose, we first detailed recovery dynamics of total thymic cells and TECs (EpCAM^+^ CD45^-^ TER119^-^ cells) from female mice on a 7-week-old C57BL/6 background at several time points after irradiation with a sublethal dose of γ-rays (5.5 Gy) (**Figure 1A**). Based on binding of UEA-1 lectin and expression level of Ly51, TECs were separated into subpopulation of mTECs (UEA-1^+^Ly51^–^ TECs) and cTECs (UEA-1^–^ Ly51^+^ TECs) (**Supplementary Figure 1**). As in previous studies (17), the cell number of thymocytes decreased until Day 4, and then increased to almost the same number on Day 10 as on Day 0 (**Figure 1B**). In contrast, the number of TECs decreased until Day 6 (**Figure 1C**). Moreover, the maximum recovery of TECs occurred on Day 15, but still remained below the level on Day 0 (**Figure 1C**). These recovery dynamics pertain to mTECs, but not to cTECs (**Figures 1D, E**). Thus, these data indicate that radiation mainly causes a reduction of mTEC cellularity under these conditions.

**Figure 2:**
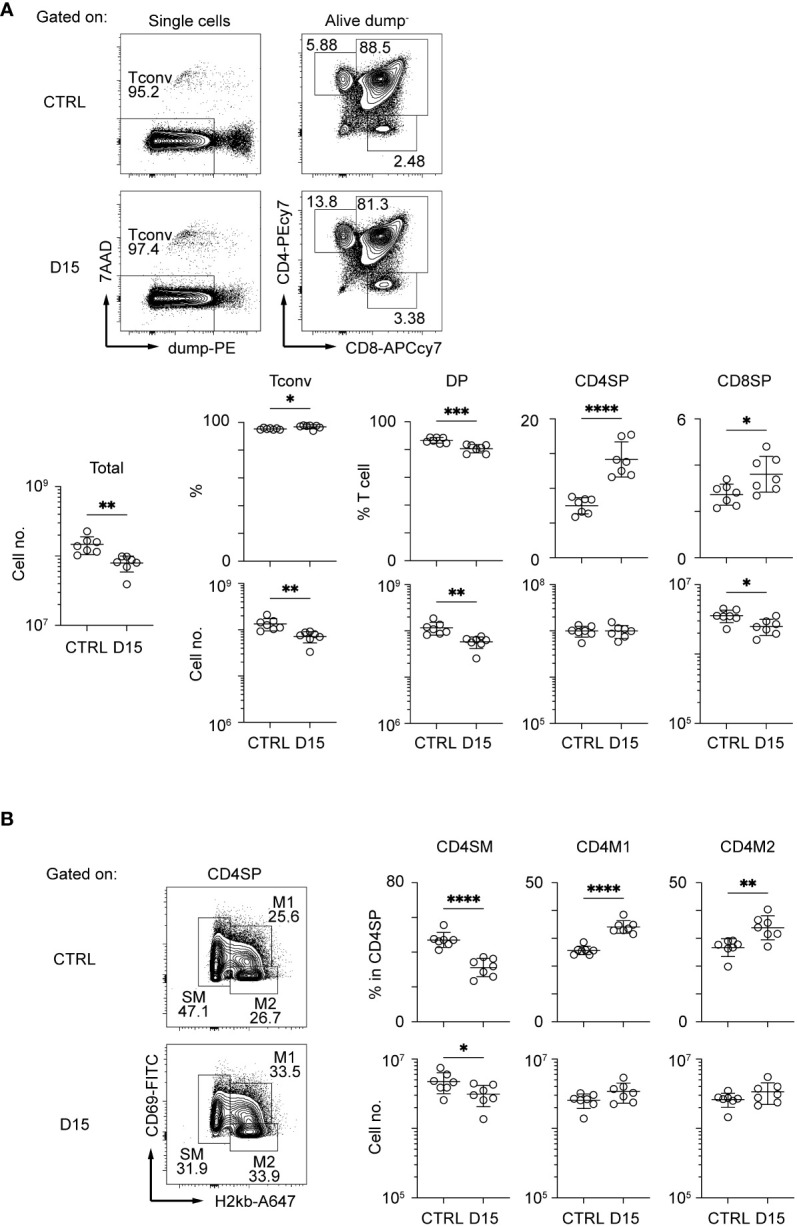
Thymocytes in the medulla were mostly recovered 15 days after sublethal irradiation. Representative flow cytometric profiles of total thymocytes **(A)** and mature SP thymocytes **(B)**. Dump^–^ cells are defined as 7AAD^–^ CD25^–^ CD44^–^ γδTCR^–^CD1d-tetramer^–^ cells. Data are summarized in figures below. Tconv indicates Dump- cells. DP, CD4SP, and CD8SP indicates CD4^+^CD8^+^Dump^–^, CD4^+^CD8^–^Dump^–^, and CD4^–^CD8^+^Dump^–^ cells *p < 0.05; **p < 0.01; ***p < 0.001; ****p < 0.0001; two-tailed t test.

Interaction between TECs and thymocytes is critical for development and regeneration of the thymus (15). Because mTEC development is dependent on TNF cytokine family signaling from CD4SP thymocytes (22, 23), we detailed aftereffects of radiation on CD4SP thymocytes. CD4SP and CD8SP thymocytes among thymic naïve T cells were separated according to the gating strategy described in a previous study (24) (**Figure 2A** and **Supplementary Figure 2A**). After irradiation, CD4SP thymocyte numbers recovered by Day 15, whereas numbers of DP and CD8SP thymocytes were slightly reduced. CD4SP thymocytes are divided into three sub-populations at different maturation stages, based on expression levels of H2-kb and CD69 (24) (**Figure 2B** and **Supplementary Figure 2B**). Thus, CD69^hi^ H2-kb^lo^ cells and semi mature cells (SM) are the most immature types of CD4SP thymocytes. CD69^lo^ H2kb^hi^ cells, type 2 mature cells (M2) are the most mature cells, and CD69^hi^ H2kb^hi^ cells, type 1 mature cells (M1), are considered an intermediate differentiation stage between SM and M2 cells (24). Ratios of SM in CD4SP were decreased and those of M1 and M2 were increased in the thymus on Day 15. Cell number was slightly decreased for SM1, but not changed for M1 and M2. Thus, development of CD4SPs had largely recovered by Day 15. Moreover, the RANKL expression and CD40L expression are rather up-regulated on Day 9 and Day 14, respectively (**Supplementary Figure 2C**). Overall, the reduction of mTECs may not simply be due to reduced cellularity and cytokine expression of CD4SP thymocytes.

### Differentiation stages from TA-mTECs to Aire^+^mTEC were affected 15 days after irradiation

**Figure 3:**
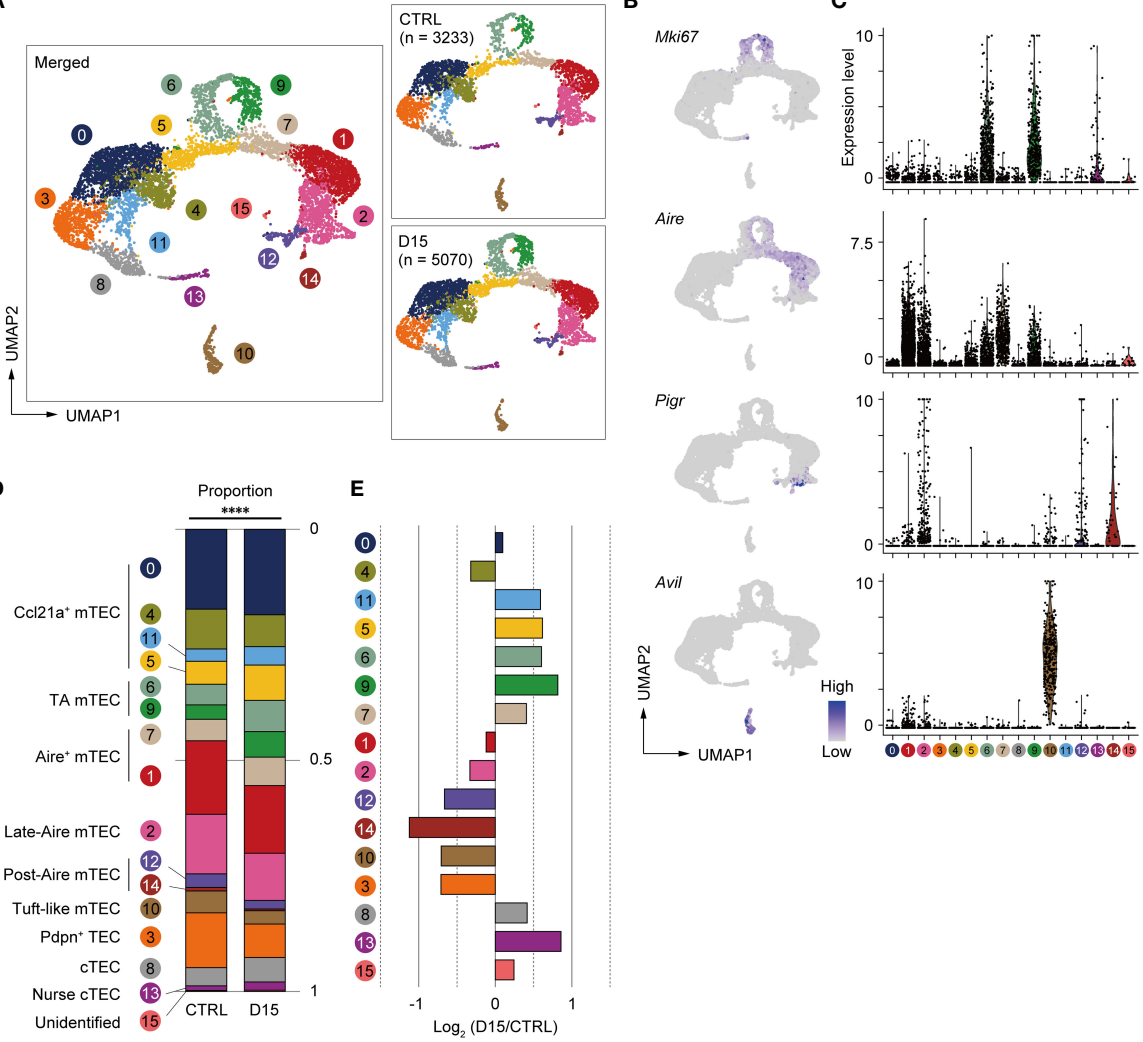
Heterogeneity of mTECs remained altered 15 days after sublethal irradiation. **(A)** UMAP plots of scRNA-seq data of TECs from mice 15 days after irradiation (D15) and age-matched control mice (CTRL). After being integrated to eliminate a batch effect, two datasets are split (right figures). Parentheses indicate cell numbers used for analysis after filtering to remove low-quality data. Cluster numbers are indicated. **(B)** Heatmaps of marker gene expression (counts per 10^4^ counts) projected onto UMAPs. **(C)** Violin plots of marker gene expression (scaled counts per 10^4^ counts) **(D)** Stacking bar plots of proportions of each cluster in TECs from mice 15 day after irradiation (D15) and age-matched control mice (CTRL). ****p < 0.0001; Chi-square goodness of fit test. **(E)** Bar plots of log2-transformed changes in proportions of TEC clusters in mice 15 day after irradiation (D15) and age-matched control mice (CTRL). Wilcoxon rank sum test. SST, steady-state thymus; PIT, post-irradiation thymus.

We next sought to find effects of irradiation on heterogeneity and transcriptomics of TECs. We performed scRNA-seq of TECs from mice 15 days after irradiation and age-matched control mice because the number of TECs and mTECs was largely recovered by this time. TECs (CD45^–^TER119^–^EpCAM^+^ cells) were sorted from collagenase-digested thymic cells of these mice for scRNA-seq. To normalize individual variation, TECs from 3 mice were pooled for each condition. TEC data from irradiated and control mice were integrated and compared using the Seurat package (25). After filtering out cells with poor-quality expression data, 8,303 cells were used for downstream analysis. Among selected cells, 5,070 cells were irradiated TECs and 3,233 cells were control TECs. Unsupervised graph-based clustering and UMAP dimension reduction of scRNA-seq data separated TECs into 16 clusters (**Figure 3A**). Based on expression levels of TEC marker genes (**Figures 3B, C** and **Supplementary Figures 3A, B**), we assigned clusters 0, 4, 5 and 11 to *Ccl21a* ^+^ mTECs, clusters 1 and 7 to *Aire* ^+^ mTECs, cluster 10 to tuft-like mTECs, clusters 12 and 14 to Post-Aire mTECs, cluster 8 to cTECs, cluster 13 to nurse cTECs, and cluster 3 to probable Pdpn^+^ TECs (26). TA-mTECs are concordant with clusters 6 and 9, which are separated most likely because of differences in cell cycle phase proportions (**Supplementary Figures 3C-E**). Cluster 2 seemed to contain both *Aire* ^+^ and *Aire* ^-^ TECs (**Figures 3B, C**) and its average expression level of *Aire* was lower than that of cluster 1; therefore, it was assigned to Late-Aire mTECs (27).

Proportions of TEC subsets were compared between the two conditions and log2-transformed changes of proportions were determined (**Figures 3D, E**). Chi-square goodness of fit tests showed a significant difference in the proportion of TEC subsets between irradiated and non-irradiated cells (**Figure 3D**). Proportions of Late-Aire mTEC, Post-Aire mTECs, tuft-like mTECs, and Pdpn^+^TECs were reduced (**Figure 3E**). In contrast, proportions of CCL21a^+^ mTECs, TA-mTECs, and cTECs were increased. In Aire^+^ mTECs, cluster 1 was slightly decreased. In contrast, cluster 7, which is a transition stage from TA-mTECs to Aire^+^mTECs (9), was increased. Overall, these data suggested that sublethal-dose radiation caused abundant aftereffects in Aire^+^ mTECs and their progeny, i.e. Late-Aire, Post-Aire, and tuft-like mTECs, more than TA-TEC progenitors and CCL21a^+^ mTECs.

We considered two possible explanations for the effect of radiation on the ratio of Aire^+^mTECs and their later differentiation stages. The first is that irradiation may promote a decrease in fully differentiated Aire^+^mTECs, which are post-mitotic and are susceptible to cell death (28). The second is that differentiation from TA-mTEC progenitors into Aire^+^mTECs was repressed by irradiation.

**Figure 4:**
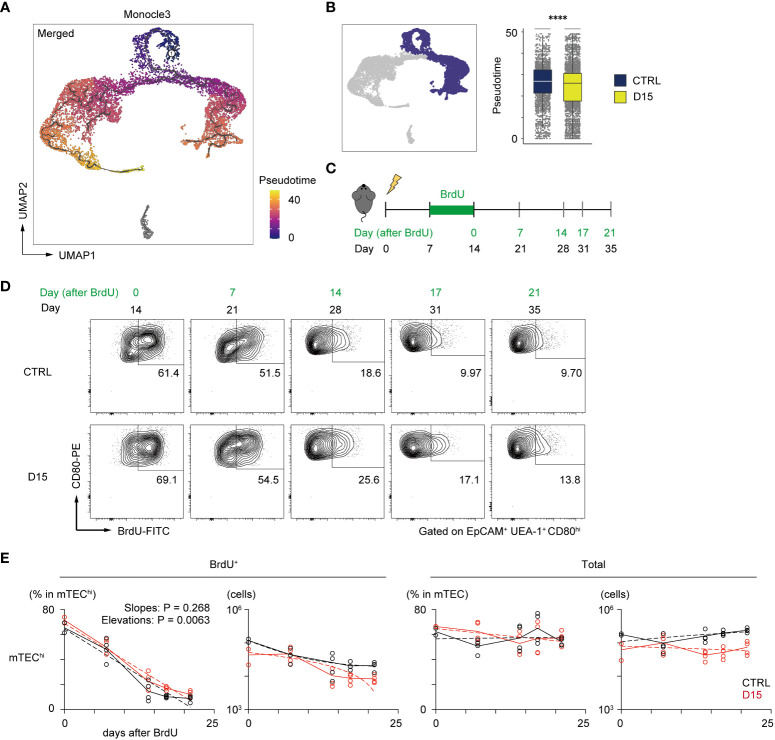
Irradiation did not influence the reduction kinetics of mature mTEC frequency. **(A)** The trajectory graph and pseudotime projected onto the UMAP plot of scRNA-seq data of TECs. **(B)** Box plots show pseudotime means of TA-mTECs, Aire^+^mTECs, and Late-Aire mTECs from mice 15 day after irradiation (D15) and age-matched controls (CTRL). Each dot indicates the pseudotime of an individual cell. ****p < 0.0001; Wilcoxon rank sum test. **(C)** A schematic diagram of *in vivo* BrdU-labeling experiment. BrdU was supplied in drinking water from day 7 to day 14 after irradiation. **(D)** Representative flow cytometer plots of mTEC^hi^ incorporating BrdU. mTEC^hi^ were defined as CD80^hi^EpCAM^+^ UEA1^+^ CD45^–^ TER119^–^cells **(E)** Decay kinetics of BrdU-labeled cells after the end of BrdU labeling. Solid lines connect means at each timepoint. Broken lines in plots of percentage against days indicate simple linear regression of average values. Broken lines in plots of cellularity were fitted by the least square method.

A previous report suggested that pseudotime analysis may predict the differentiation rate of cells (29). We then performed single-cell trajectory analysis of TEC scRNA-seq data using the Monocle 3 package (30) to determine the pseudotime of each cell. Cells closest to TA-mTECs were chosen as root cells, which is the starting point for the pseudotime trajectory (**Figure 4A**). To evaluate differences in the differentiation rate, the pseudotime mean of cells around the branching of TA-mTEC, Aire^+^mTEC and Late Aire mTEC stages was calculated for control and irradiated TECs. The pseudotime mean of TECs receiving radiation was significantly lower than that of control TECs (**Figure 4B**), implying that the differentiation rate of mature mTECs is slowed 15 days after irradiation.

In order to evaluate reduction kinetics of differentiated mTECs, we performed a 5-bromo-2’-deoxyuridine (BrdU)-labeling assay. BrdU labeling of TA-mTECs and differentiated mTECs was performed by supplying BrdU in drinking water during the recovery period (Days 7 to 14 in **Figure 4C**). After BrdU labeling, the frequency of BrdU-labeled mTECs (UEA-1^+^EpCAM^+^) was monitored by flow cytometric analysis for 3 weeks (**Figure 4D**). Immediately after the end of the BrdU up-take, the ratio of BrdU-labeled cells in mTECs expressing high levels of CD80 (mTEC^hi^), which include TA-mTECs, Aire^+^mTECs and Post-Aire mTECs, was 69.1% of irradiated mTEC^hi^, versus 61.4% of the control group, suggesting similar proliferation of mTEC^hi^ in the two conditions. As expected, the proportion of BrdU-labeled cells in mTEC^hi^ gradually decreased and plateaued by Day 17 after BrdU-labeling, whereas total TEC numbers were practically constant during this period (**Figure 4E**). Notably, reduction kinetics of BrdU-labeled mTEChi were similar between the two conditions (**Figure 4E**). Thus, while it is possible that mTEC^hi^ is susceptible to apoptosis immediately after irradiation, these results suggest that the sensitivity of mTEC^hi^ to cell death might not be largely different between irradiated and non-irradiated thymus at 15 days post-irradiation. Accordingly, it appears that the reduction in the frequency of mTEC^hi^ at Day 15 is likely due to a repression in mTEC differentiation during the recovery period, and/or preferential cell death of mTEC^hi^ early after irradiation.

### Expression levels of tissue-specific antigens were altered by irradiation

**Figure 5:**
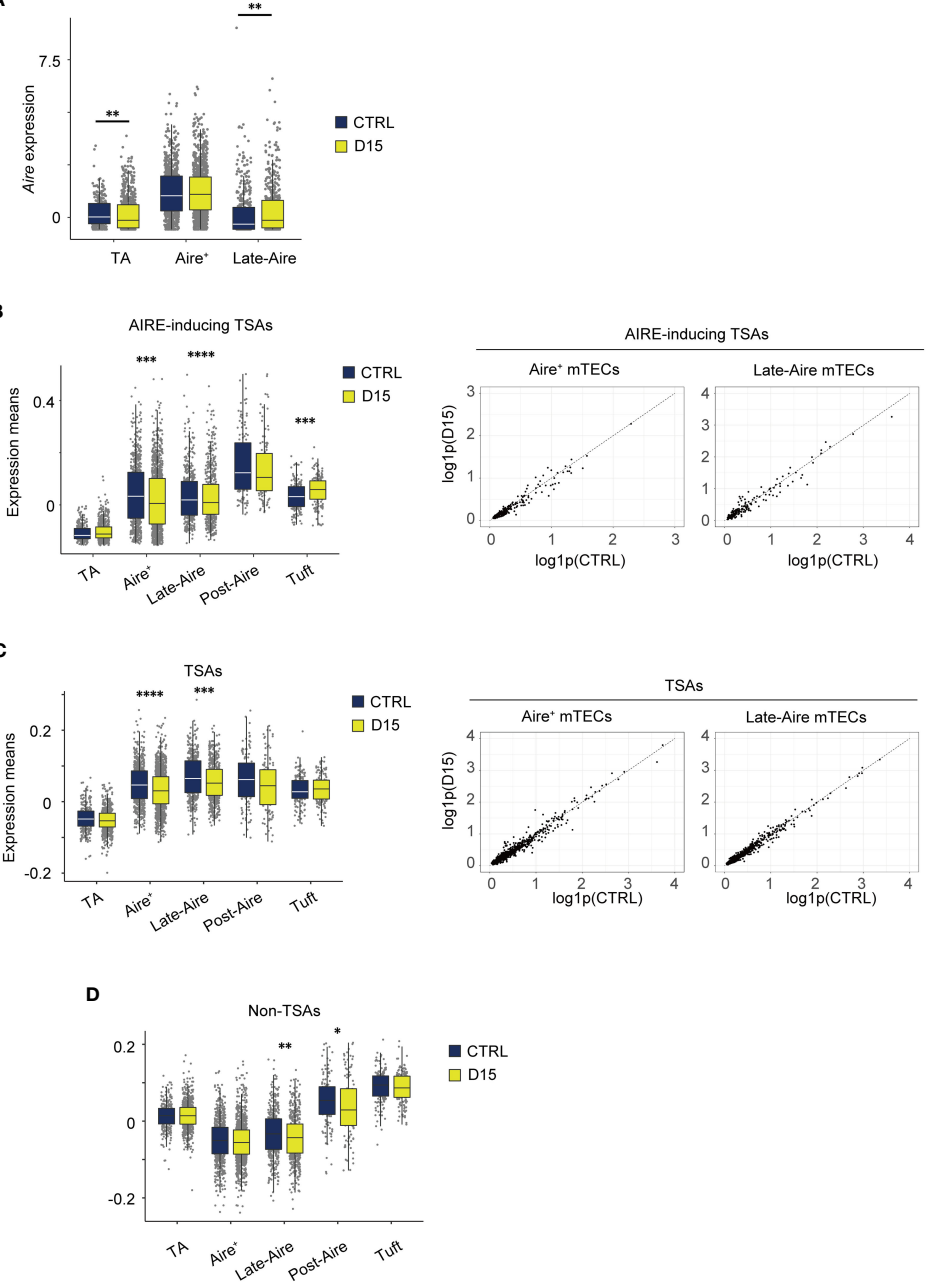
Expression level of AIRE and tissue-specific antigen genes were altered 15 days after acute sublethal irradiation. **(A)** A box plot of means of *Aire* expression in indicated cell subsets (TA, TA-mTECs, Aire^+^, Aire^+^mTECs, and Late-Aire, Late-Aire mTECs) from mice 15 days after sublethal irradiation (D15) and controls (CTRL). **(B-D)** Box plots for mean TSA expression, AIRE-inducing TSAs and non-TSAs from mice 15 days after irradiation (D15) and controls (CTRL). TA, Aire^+^, Late-Aire and Tuft indicate TA-mTECs, Aire^+^mTECs, Late-Aire mTECs, and tuft-like mTECs. TSA and Aire-inducing TSAs were selected according to a previous study (31). *p < 0.05; **p < 0.01; ***p < 0.001; ****p < 0.0001; Wilcoxon rank sum test. For gene expression analysis, genes with more than 500 UMI counts were selected as practically expressed genes.

We next investigated irradiation-induced aftereffects in gene expression of mTECs, using scRNA-seq data. We focused on gene expression changes in Aire^+^mTECs and their later differentiation stages because of their greater susceptibility to radiation. *Aire* expression was highest in Aire^+^ mTECs, comprising clusters 1 and 7, and moderate in Late-Aire mTECs (cluster 2) and TA-mTECs comprising clusters 6 and 9 in control mice (**Figure 5A**). Radiation caused a slight down-regulation of *Aire* expression in TA-mTECs and up-regulation of Late-Aire mTECs at Day 15, whereas *Aire* expression was not significantly altered in Aire^+^mTECs (**Figure 5A**).

Because Aire expression was differentially altered, we next investigated aftereffects on TSA expression in each subset. A list of TSAs and AIRE-inducing TSAs (**Supplementary Table 1**), which are down-regulated by *Aire* gene deletion (31), were used for this analysis. Mean expression levels of all TSAs and AIRE-inducing TSAs were slightly reduced in Aire^+^mTECs and Late-Aire mTECs from irradiated mice, compared to control mice (**Figures 5B, C**) although changes in individual TSA expression level are small (**Figures 5B, C**). Interestingly, for Late-Aire mTECs, Non-TSA genes in addition to TSA genes were reduced (**Figure 5D**), suggesting a possible effect of radiation on general gene expression mechanisms. For tuft-like mTECs, total TSA expression was unchanged and the mean expression level of AIRE-inducing TSAs was increased (**Figures 5B, C**). Interestingly, although Aire expression was unchanged in Aire^+^ mTECs and increased in Late-Aire mTECs (**Figure 5A**), mean expression levels of AIRE-inducing TSAs were decreased in both mTECs. Thus, the change in expression level of AIRE-inducing TSAs by irradiation was not correlated with the change in AIRE expression level, implying that other regulatory mechanism(s) controlling ectopic TSA expression might be disturbed by irradiation.

### mTECs expressing Ccl25 were selectively decreased 15 days after irradiation

**Figure 6:**
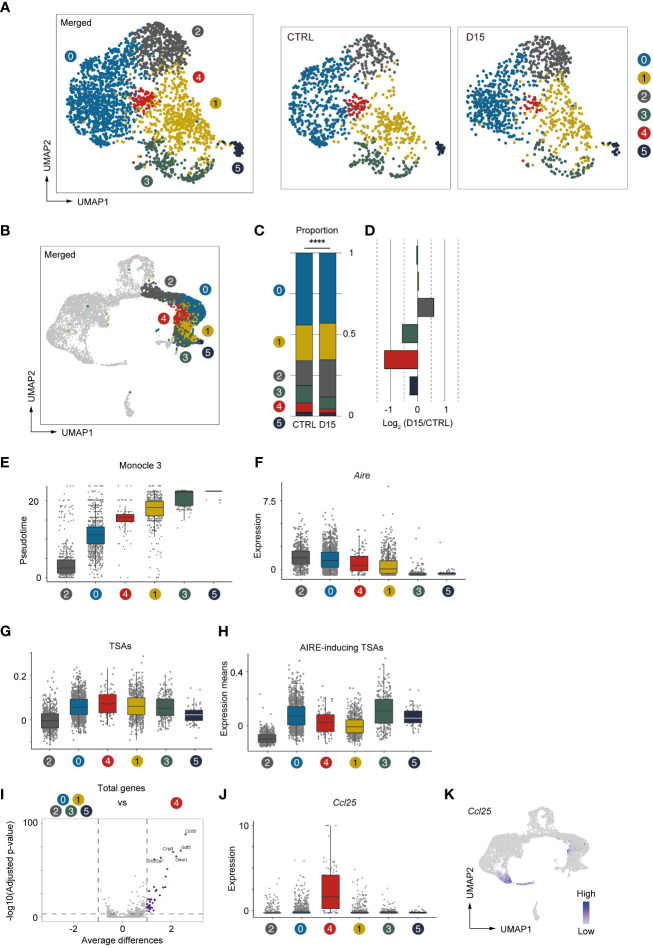
*Aire* ^+^ mTECs expressing CCL25 and a high level of TSAs were preferentially decreased 15 days after acute irradiation. **(A)** UMAP plot for subclustering analysis of Aire^+^mTECs and Late-Aire mTECs. Only TSA genes were used for subclustering and UMAP reduction. **(B)** Embedding clusters of **(A)** in the original UMAP plot using all cells and genes. **(C)** Stacking bar plots of proportions of each subcluster from TECs mice 15 day after sublethal irradiation (D15) and controls (CTRL). ****p < 0.0001. **(D)** Bar plots of log2-transformed changes in proportions of TEC subclusters from mice 15 day after irradiation (D15) and age-matched control mice (CTRL) **(E)** Box plots shows pseudotimes of cells in subclusters determined based on TSA expression. Each dot indicates a pseudotime of an individual cell. Box plots of *Aire* expression **(F)**, means of AIRE-inducing TSA expression **(G)**, and means of TSA expression **(H)** in each subcluster are indicated. **(I)** A volcano plot of changes in gene expression between subcluster 4 and other subclusters. Purple dots indicate significantly upregulated genes in subcluster 4 (p-value < 0.05, average differences > 1). P-values were calculated with the Wilcoxon rank-sum test with Bonferroni correction. **(J)** Box plots of *Ccl25* expression in each subcluster. **(K)** A heatmap of Ccl25 expression projected onto UMAP. Gene expression values were scaled counts per 10^4^ counts.

We next conducted a detailed analysis of Aire^+^mTECs and Late-Aire mTECs, which are considered mature mTECs expressing high levels of MHC class II and co-stimulatory molecules. Because the TSA expression profile is altered by irradiation, we performed subclustering and UMAP dimension reduction based on TSA expression levels for these mTECs. We found that Aire^+^mTECs and Late-Aire mTECs are separated into 6 subclusters by expression levels of TSAs (**Figure 6A**). Embedding these subclusters in the original UMAP plot suggested that subclusters 0 and 2 are mainly concordant with Aire^+^mTECs and that subclusters 1, 3, 4, and 5 are concordant with Late-Aire mTECs (**Figure 6B**). Comparisons of the proportions of these subclusters between irradiated TECs and controls revealed that subcluster 4 was most severely decreased by irradiation (**Figures 6C, D**). Subcluster 4 is located close to the border between Aire^+^mTECs and Late-Aire mTECs in the original UMAP plot (**Figure 6B**). Consistently, Monocle 3 analysis suggested that the average pseudotime of cluster 4 cells is between that of cluster 0 and cluster 1 (**Figure 6E** and **Supplementary Figure 3F**), which are typical clusters of Aire^+^mTECs and Late Aire mTECs, respectively. Moreover, expression of Aire in subcluster 4 is intermediate between those of clusters 0 and 1 (**Figure 6F**). Thus, this radiation-sensitive subcluster 4 most likely corresponds to an intermediate subset between Aire^+^mTECs and Late-Aire mTECs.

Because subcluster 4 was detected by subclustering and UMAP analysis based on TSA expression, we analyzed TSA expression in subcluster 4 (**Figure 6G**). Interestingly, expression levels of TSAs are highest among these subclusters. However, expression of Aire-inducing TSAs was moderate and lower than that of subcluster 0 (**Figure 6H**). This finding implies that the profile of TSAs expressed in mature mTECs is dynamically changed during maturation. We next sought to find marker genes specific to subcluster 4. Differential gene expression analysis of subcluster 4 and other subclusters revealed significant up-regulation of several genes in cluster 4 (**Figure 6I**). In particular, C-C motif chemokine ligand 25 gene (*Ccl25*) is most differentially expressed in cluster 4 (**Figures 6I, J**). Thus, the analysis suggested that, in addition to cTECs, a specific subset of Aire^+^mTEC highly expresses CCL25 (**Figure 6K**). This CCL25^+^ cell cluster appears to be present in other datasets of mTECs (11, 12) (**Supplementary Figure 4**). Importantly, these CCL25-expressing mTECs may be decreased after acute irradiation.

## Discussion

In this study, we investigated aftereffects of sub-lethal dose irradiation on murine TEC phenotypes. Data suggested that even if the number of total thymic cells recovered, recovery of mTEC cellularity was not complete. scRNA-seq analysis suggested that reduction occurs in Aire^+^mTECs and mTECs in later differentiation stages, which are considered mature mTECs. Importantly, we found that the frequency of TA-mTEC progenitors was less influenced by radiation, compared to mature mTECs. Label retention experiments suggested that the reduction in cellularity of mature mTEC subsets was not due to enhanced mTEC cell death, but may be due to decelerated differentiation from TA-mTECs to Aire^+^mTECs. The important question is what mechanisms of differentiation are affected by irradiation.

TNF family RANKL signaling is critical for differentiation of Aire^+^mTECs (22, 32, 33). Moreover, a previous study revealed that RANKL is up-regulated in CD4SP and type 3 ILCs after bone marrow transplantation with irradiation, and the administration of recombinant RANKL protein boosts the regeneration of mTECs (34). On the other hand, a recent study suggested that RANKL signaling may promote differentiation from immature mTECs to TA-mTECs (8). It should be noted that the ratio of TA-mTECs was higher than that of controls. Accordingly, it is unlikely that impairment of RANKL signaling is solely responsible for reduction of Aire^+^ mTECs and their descendants. This idea may be supported by the fact that CD4SP cells were significantly rescued 15 days after irradiation, and their RANKL expression was rather increased following irradiation (**Supplementary Figure 2C**). Thus, it is an open question to identify the mechanism by which irradiation causes a selective effect on development of these mTECs.

One previous study revealed a critical role of IL-22 for TEC regeneration after irradiation (16). IL-22 is expressed in type 3 innate lymphoid cells via signaling of IL-23, expressed in dendritic cells sensing the reduction of CD4 CD8 double-positive thymocytes. This mechanism promotes proliferation of TECs in relatively early stages of thymic recovery. Thus, it is likely that the IL-22-dependent event occurs ≤15 days after irradiation. However, given that IL-22 signaling enhances proliferation of TECs, most likely including proliferative TA-mTECs, it may be that TA-mTECs receiving IL-22 signaling are less able to differentiate into Aire^+^ mTECs. Reduction of Aire expression in TA-mTECs after irradiation suggests that properties of TA-mTECs can be changed by irradiation. To test this hypothesis, it is necessary to compare phenotypes of irradiated and non-irradiated TA-mTECs in detail. However, presently, it is difficult to discriminate between TA-mTECs and Aire^+^mTECs by flow cytometry. Identification of a cell surface marker for TA-mTECs will help overcome this problem.

One surprising finding in our scRNA-seq analysis is that an mTEC subset expressing high levels of CCL25 is present in the Late-Aire mTEC cluster. CCL25 is highly expressed in cTECs and controls recruitment of T cell progenitors derived from bone marrow into the thymus (35). Interestingly, a recent study suggested that CCL25 is directly regulated by AIRE (36). Moreover, the same authors reported that CCL25-deficient mice showed autoimmunity in some organs, which was similar to the phenotype of *Aire*-deficient mice. Therefore, in addition to detailed characterization, an important future study should address whether CCL25-expressing mTECs are critical to suppress onset of autoimmunity.

Recent studies have demonstrated that post-Aire mTECs can be separated into some mimetic cell subsets, which exhibit gene expression patterns resembling those of various peripheral tissues (11, 12). In contrast, the CCL25+ mTECs shown in this study are a subset at the boundary between Aire+ mTECs and Late-Aire mTECs and are marked by elevated expression of various tissue-specific genesConsequently, it is conceivable that Aire^+^ mTECs may also subdivide into minor cell subsets with slightly distinct gene expression profiles. While a previous single-cell RNA-sequencing analysis has hinted at the existence of these minor subsets (5), their experimental validation remains an important aspect to be addressed.

One limitation of this study is that only one experiment was conducted for scRNA-seq analysis of irradiated and control samples. Therefore, efforts were made to minimize individual differences among mice by pooling TECs from three mice. Furthermore, a comparison of the integrated data under these two conditions suggests that the batch effect due to experimental error is small. However, future validation of the present scRNA-seq data is needed through flow cytometry analysis of cell subsets with appropriate cell surface markers and RNA-seq analysis of sorted cell subsets.

It was reported that radiation increases the risk of autoimmune diseases, such as diabetes and thyroid autoimmune disease (37, 38)). Whereas most studies have focused on the dysregulation of peripheral lymphocytes, the disturbance of the thymic self-tolerance mechanism may also contribute to the increased risk of autoimmune diseases after exposure to radiation through radiotherapy or disasters. Indeed, a recent study showed that the recovery of mTEC numbers was delayed compared to mature thymic T cells after bone marrow transplantation in mice, leading to autoimmunity (39). Our findings may further support this, as we observed a decrease in the percentage of mature mTECs in the total TECs 15 days after total-body irradiation, despite the recovery of CD4SP cells. In addition, the expression level of TSA in mature mTECs was also decreased. Thus, these two effects on mTECs lead to the impairment of the thymic medullary environment responsible for self-tolerance after irradiation and may combine to increase the risk of autoimmunity. Further analysis of the variation in the TCR repertoire of mature thymic T cells after irradiation may provide some insight into this issue.

Overall, our study suggests an impact of irradiation on properties and heterogeneity of mTECs, which may disrupt thymic self-tolerance mechanisms, thereby increasing the risk of autoimmune diseases. Further verification of this hypothesis is important for discovering mechanisms underlying increased risk of autoimmune disease onset due to irradiation and other thymic insults.

## Materials and methods

### Mice

All mice were housed under specific pathogen-free conditions according to Guidelines of the Institutional Animal Care and Use Committee of RIKEN, Yokohama Branch (2018- 075). C57BL/6 female mice (Clea, Japan) were used for all experiments. Seven-week-old female mice received 5.5 Gy of γ-irradiation. For scRNA-seq analysis, and flow cytometric analysis of thymocytes, mice were used 15 days after irradiation. For BrdU labeling, 0.8 mg/mL BrdU (Nacalai, Kyoto, Japan) was supplied with sterile drinking water for 7 days.

### Antibodies and reagents

Antibodies and reagents employed included APCCy7-anti-mouse CD45 (BioLegend, clone 30 F-11, Cat#103116), APCCy7-anti-mouse TER119 (BioLegend, clone TER-119, Cat#557853), BV711-anti-mouse EpCAM (CD326) (BioLegend, clone G8.8, Cat#118233), PE-anti-mouse CD80 (eBioscience, clone 16-10A1, Cat#12-0801-81), Alexa647-anti-mouse Ly51 (BioLegend, clone 6C3, Cat#108312), Biotinylated UEA-1 (Vector Laboratories, Cat#B-1065), PECy7-streptavidin (eBioscience, Cat#25-4317-82), PECy7-anti-mouse CD4 (BioLegend, clone RM4-5, Cat#100528), APCCy7-anti-mouse CD8 (BioLegend, clone 53-6.7, Cat#109220), PE-anti-mouse CD44 (BioLegend, clone IM7, Cat# 103007), PE-anti-mouse CD25 (BioLegend, clone PC61, Cat#102007), PE-anti-mouse TCRγ/δ (BioLegend, clone GL3, Cat#118107), FITC-anti-mouse CD69 (BioLegend, clone H1.2F3, Cat#104506), AlexaFluor647-anti-mouse H2-kb (BioLegend, clone AF6-88.5.5.3, Cat#116512), FITC-anti-BrdU (BD, BrdU flow kit, Cat#559619), Zombie Aqua fixable viability kit (BioLegend, Cat#423101), and anti-mouse CD16/32 (BioLegend, clone 93, Cat#101302).

### Isolation and analysis of TECs from mice

Mice were sacrificed with CO_2_ and thymi were dissected. Individual thymi were minced with razor blades and thymic fragments were obtained by removing the supernatant and by pipetting minced thymi up and down in 1 mL RPMI1640 (Wako). Thymic fragments were digested in 0.05U/mL Liberase (Roche) and 0.01%w/v DNaseI (Sigma-Aldrich) in RPMI1640 by incubation at 37°C 3 x 12 min. Supernatant from the digestion was added to the same volume of FASC buffer (2% fetal bovine serum; FBS in phosphate-buffered saline; PBS) containing 1 nM EDTA to stop reactions. The cell suspension was incubated with anti-mouse CD16/32 in FACS buffer to block non-specific binding, and stained with primary antibodies (anti-EpCAM, -CD45, -TER119) in FACS buffer. 7-Amino-Actinomycin D (7-AAD) was added to label dead cells. EpCAM^+^ cells were sorted for scRNA-seq using a FACS Aria instrument (BD). For BrdU detection, cells were stained with primary antibodies (anti-EpCAM, -Ly51, -CD45, -TER119, -CD80, biotinylated UEA-1) in FACS buffer and sequentially incubated with secondary reagent (streptavidin) in FACS buffer following a blocking step. Dead cells were labeled with Zombie Aqua fixable viability kit. Flow cytometry data were analyzed with FlowJo10.

### BrdU-labeling experiment

BrdU up taken in DNA was detected using a BrdU flow kit (BD, Cat#559619). In brief, cells were fixed and permeabilized with Cytofix/Cytoperm buffer after cell surface staining. To expose BrdU up taken by DNA, cells were incubated with 300mg/mL DNaseI in DPBS for 1 h at 37°C before anti-BrdU staining.

### Library preparation for droplet-based single-cell RNA-sequencing

For scRNA-seq analysis, cell suspensions of steady-state thymi or post-irradiated thymi from three mice were prepared and pooled. Cellular suspensions were loaded onto a Chromium instrument (10× Genomics) to generate a single- cell emulsion. scRNA-seq libraries were prepared using Chromium Single-Cell 3′ Reagent Kit v3 Chemistry.

### scRNA-seq analysis of TECs

Library paired-end sequencing was performed on an Illumina HiSeq2000. Output FASTQ files were processed using Fastp, demultiplexed, and aligned to the mm10 reference genome with Cell Ranger v5.0.1. Processing with Cell Ranger was performed on the HOKUSAI supercomputer at RIKEN. Downstream analyses were performed with the Seurat package, version 4. Transcript-by-cell metrics were made from output files and contained genes expressed in at least five cells. Cells with a percentage of mitochondrial transcripts < 15% and 7500 > the number of feature RNA > 2000 were used for further analyses. For normalization, feature counts of each cell were divided by total counts of that cell and were multiplied by 10^4^. The 3000 most variable genes were selected with variance stabilizing transformation to perform principal component analysis (PCA). After integration of two data sets with 2000 anchor features, scaling data, PCA and UMAP, followed by shared-nearest-neighbor graph construction were performed. Shared-nearest-neighbor graph construction was performed with the top 20 PCs and resolution of cell clustering analysis was set at 0.5. Subclustering analysis was performed with only TSA genes defined as a list (**Supplementary Table 1**). Cluster-based differential gene expression analysis was performed using FindMarkers in the Seurat package with scaled expression values. Pseudotime analysis was performed with the Monocle3 package.

### Reanalysis of published single-cell and single-nucleus RNA-seq datasets of mTECs

Two published datasets of single-cell (11) or single-nucleus (12) RNA-seq data of mTECs were reanalyzed to confirm reproducibility of the Ccl25-expressing mTEC population we discovered by integration with our scRNA-seq data. In reanalysis of a scRNA-seq dataset from Michelson et al, we obtained fastq files of multiplexed samples of CD104^lo^ mTEC^lo^ from wild-type adult and perinatal mice (GSE194253), demultiplexed, and aligned to the mm10 reference genome with Cell Ranger v5.0.1 on NIG supercomputer. Metrics according to hash-tags were also obtained from NCBI (GSE194253). Preprocessing prior to integration analysis was performed as previously described (11). Briefly, cells without hash-tags, cells with multiple hash-tags, and cells with more than 20% mitochondrial RNA occupying total RNA were removed as low quality cells and read counts were normalized by dividing feature counts of each cell by total counts oh that cell and then multiplied by 10^4^. In reanalysis of a snRNA-seq dataset from Givony et al, we obtained fastq files (GSE194253), followed by the same processes as scRNA-seq datasets. In preprocessing prior to integration analysis, cells with less than 500 counts or more than 5000 counts of the number of feature RNA and cells with more than 15% mitochondrial RNA were removed and normalization of read counts was performed as above. After preprocessing, all datasets were integrated using the FindIntegrationAnchors function and the IntegrateData function using the 3000 most valuable genes selected by the FindVariableFeatures function. 2D UMAP visualization was performed using the top 30 PCs.

### Isolation and analysis of thymocytes

Mice were sacrificed with CO_2_ and thymi were dissected. Individual thymi were ground with glass slides in 3 mL 5% FBS in RPMI1640 (Wako). Cells were centrifuged, incubated with anti-mouse CD16/32 in FACS buffer to block non-specific binding and stained with primary antibodies (anti-CD4, -CD8, -CD69 and -H2-kb) in FACS buffer. 7-Amino-Actinomycin D (7-AAD) was added to label dead cells. Flow cytometry data were analyzed with FlowJo10.

### Statistical analysis

Statistical significance between mean values was determined using Student’s t-test or the Wilcoxon rank sum test. P-values for differences of proportions of clusters in scRNA-seq analysis were calculated using the Chi-square goodness of fit test. P-value correction in differential gene expression analysis with Seurat were performed with Bonferroni methods. P-values for BrdU decay kinetics experiment were calculated by simple linear regression in GraphPad Prism. ****: p < 0.0001; ***: p < 0.001; **: p < 0.01; *: p < 0.05.

## Data availability statement

The datasets presented in this study can be found in online repositories. The names of the repository/repositories and accession number(s) can be found below: GSE228198 (GEO).

## Ethics statement

The animal study was approved by Institutional Animal Care and Use Committee of RIKEN, Yokohama Branch. The study was conducted in accordance with the local legislation and institutional requirements.

## Author contributions

KH, Data curation, Formal analysis, Funding acquisition, Investigation, Validation, Writing – original draft; MM, Data curation; TI, Investigation, Writing – review and editing, MH, Validation; YM; Validation, Writing – original draft; TM, Supervision, Writing – review and editing; SM, Supervision, Writing – review and editing; NA, Formal analysis, Funding acquisition, Investigation, Supervision, Writing – review and editing; TA, Formal analysis, Funding acquisition, Investigation, Project administration, Supervision, Validation, Writing – review and editing.

## Supplementary material

Supplementary Figure 1 Flow cytometric analysis of the post-irradiation thymus. Representative flow cytometer plots of TECs, each day after irradiation (related to **Figure 1**).

Supplementary Figure 2 Flow-cytometric and PCR analysis of thymocytes. **(A)** Flow cytometric analysis of thymocytes 15 days after irradiation *: p < 0.05; **: p < 0.01; ***: p < 0.001; two-tailed student’s t-test. **(B)** Flow cytometric analysis of CD8SP subsets. *: p < 0.05; **: p < 0.01; ****: p < 0.0001; two-tailed student’s t-test. **(C)** Quantitative PCR analysis for expression of CD40L (left) and RANKL (right) in CD4SP. Relative expression of each genes to 36B4 is shown. *: p < 0.05. Ordinary one-way ANOVA. *: p < 0.05.

Supplementary Figure 3 Analysis of scRNA-seq data. **(A)** Heatmaps of marker gene expression (counts per 10^4^ counts) projected onto UMAP plots. **(B)** Violin plots of marker gene expression (scaled counts per 10^4^ counts). **(C)** Cell-cycle scores of individual cells. All cells were assigned in G1, G2/M, and S phase of cell cycles based on expression of cell cycle related genes, using the Seurat package. **(D)** Cell-cycle scores of clusters 6 and 9 corresponding to TA-mTECs. **(E)** Scores of S (left) and G2/M (right) were plotted for individual cells from clusters 6 and 9. **(F)** Monocle 3 trajectory and pseudotime analysis of Aire+mTECs and Late-Aire mTEC subsets. ****: p < 0.0001; Wilcoxon rank sum test.

Supplementary Figure 4 Integrative analysis of scRNA-seq data. Our scRNA-seq data (CTRL and D15) are integrated with previously reported scRNA-seq data sets. **(A)** UMAP projection of integrated data, assignment of clusters, and expression of CCL25 in cell clusters. **(B)** Typical expression of marker genes in each cluster.

Supplementary Table 1 Lists of total tissue-specific antigens (TSAs) and Aire-dependent TSAs.
