# Supplementary figures and images for: Acute irradiation causes a long-term disturbance in the heterogeneity and gene expression profile of medullary thymic epithelial cells
Source: Front Immunol. 2023 Nov 2;14:1186154. doi: 10.3389/fimmu.2023.1186154 (PMC10652284; doi:10.3389/fimmu.2023.1186154)

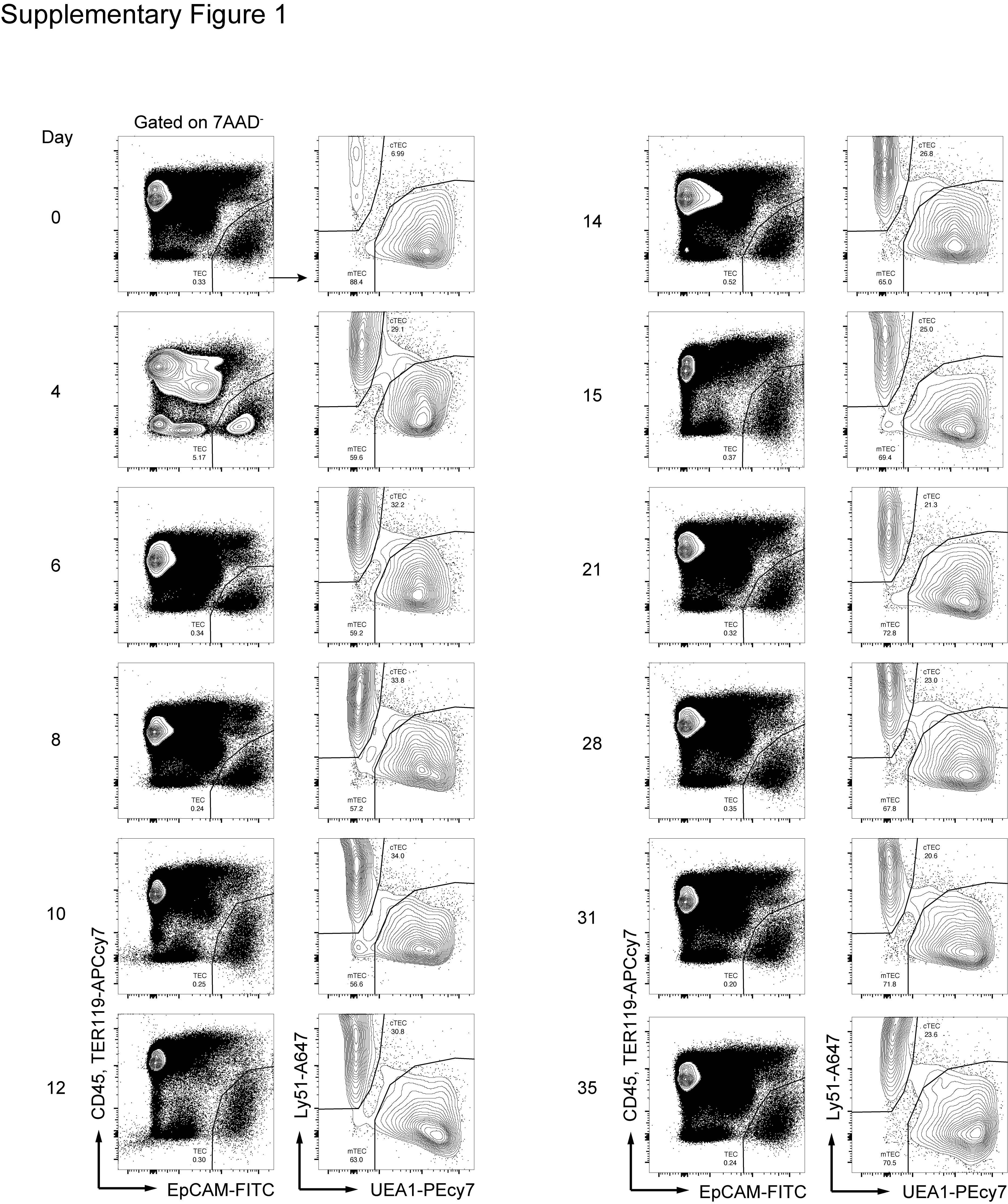

Supplement: Supplementary Figure 1 — Flow cytometric analysis of the post-irradiation thymus. Representative flow cytometer plots of TECs, each day after irradiation (related to Figure 1 ). [file Image_1.tif]

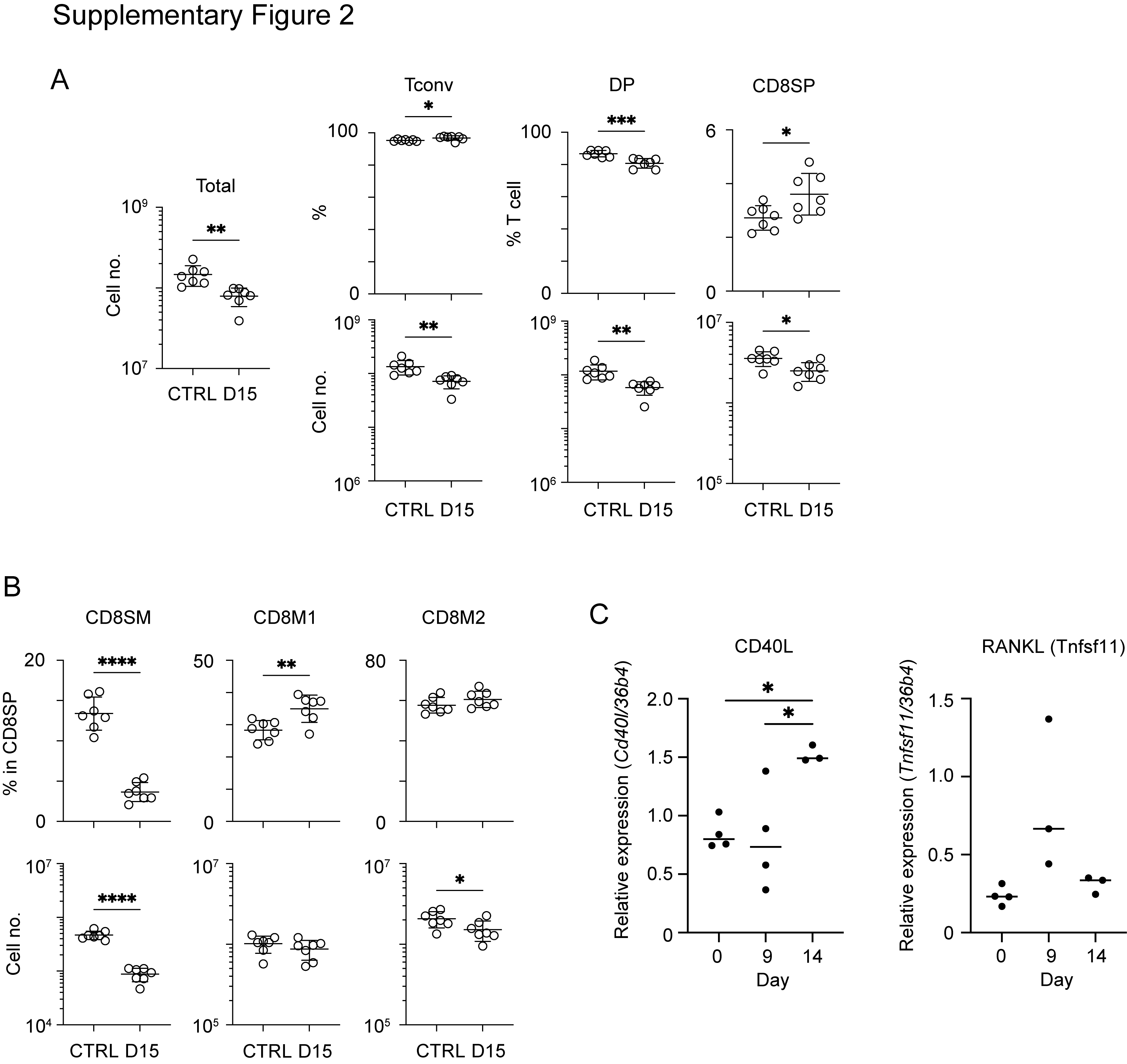

Supplement: Supplementary Figure 2 — Flow-cytometric and PCR analysis of thymocytes. (A) Flow cytometric analysis of thymocytes 15 days after irradiation *: p < 0.05; **: p < 0.01; ***: p < 0.001; two-tailed student’s t-test. (B) Flow cytometric analysis of CD8SP subsets. *: p < 0.05; **: p < 0.01; ****: p < 0.0001; two-tailed student’s t-test. (C) Quantitative PCR analysis for expression of CD40L (left) and RANKL (right) in CD4SP. Relative expression of each genes to 36B4 is shown. *: p < 0.05. Ordinary one-way ANOVA. *: p < 0.05. [file Image_2.tif]

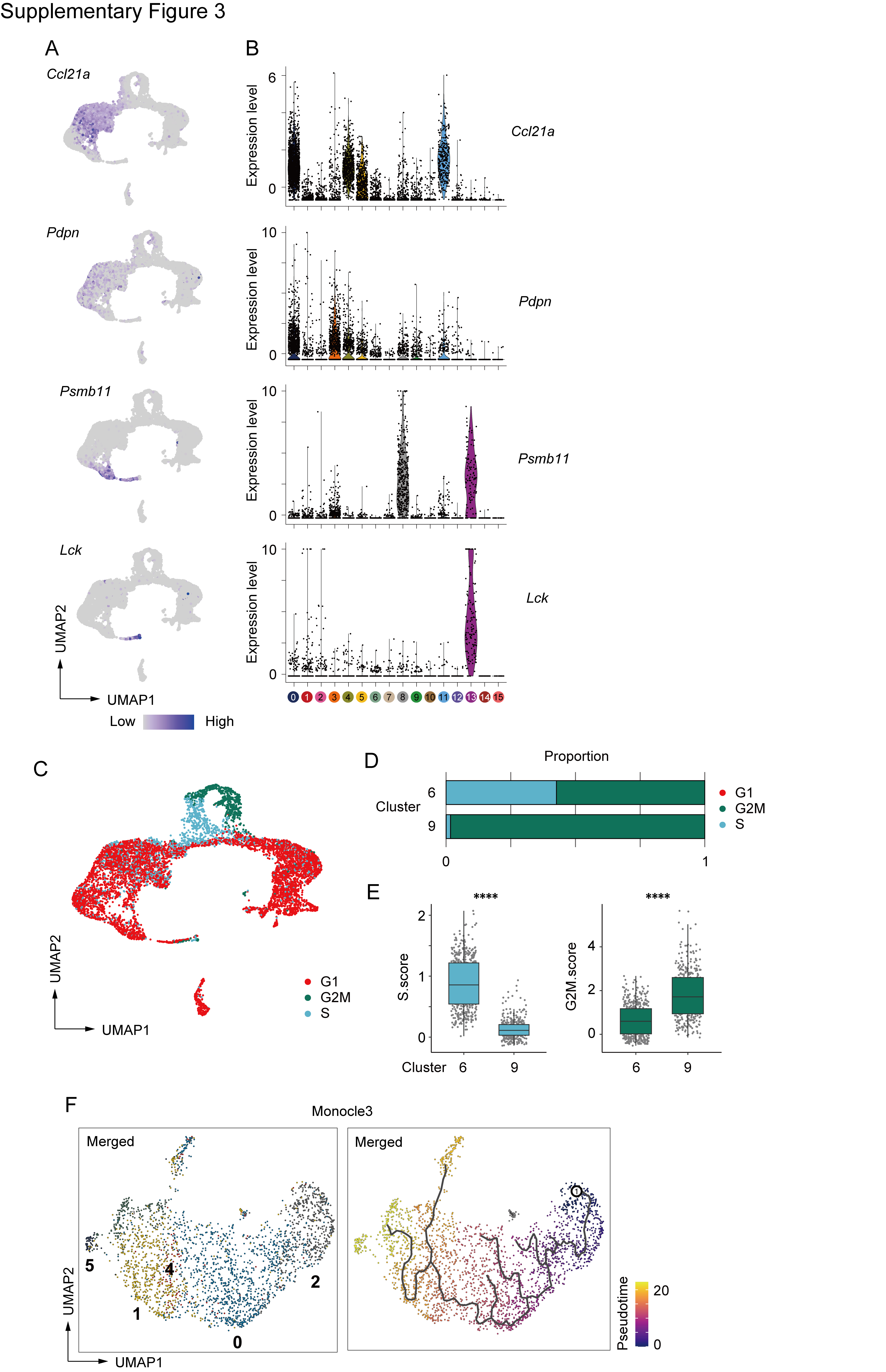

Supplement: Supplementary Figure 3 — Analysis of scRNA-seq data. (A) Heatmaps of marker gene expression (counts per 104 counts) projected onto UMAP plots. (B) Violin plots of marker gene expression (scaled counts per 104 counts). (C) Cell-cycle scores of individual cells. All cells were assigned in G1, G2/M, and S phase of cell cycles based on expression of cell cycle related genes, using the Seurat package. (D) Cell-cycle scores of clusters 6 and 9 corresponding to TA-mTECs. (E) Scores of S (left) and G2/M (right) were plotted for individual cells from clusters 6 and 9. (F) Monocle 3 trajectory and pseudotime analysis of Aire+mTECs and Late-Aire mTEC subsets. ****: p < 0.0001; Wilcoxon rank sum test. [file Image_3.tif]

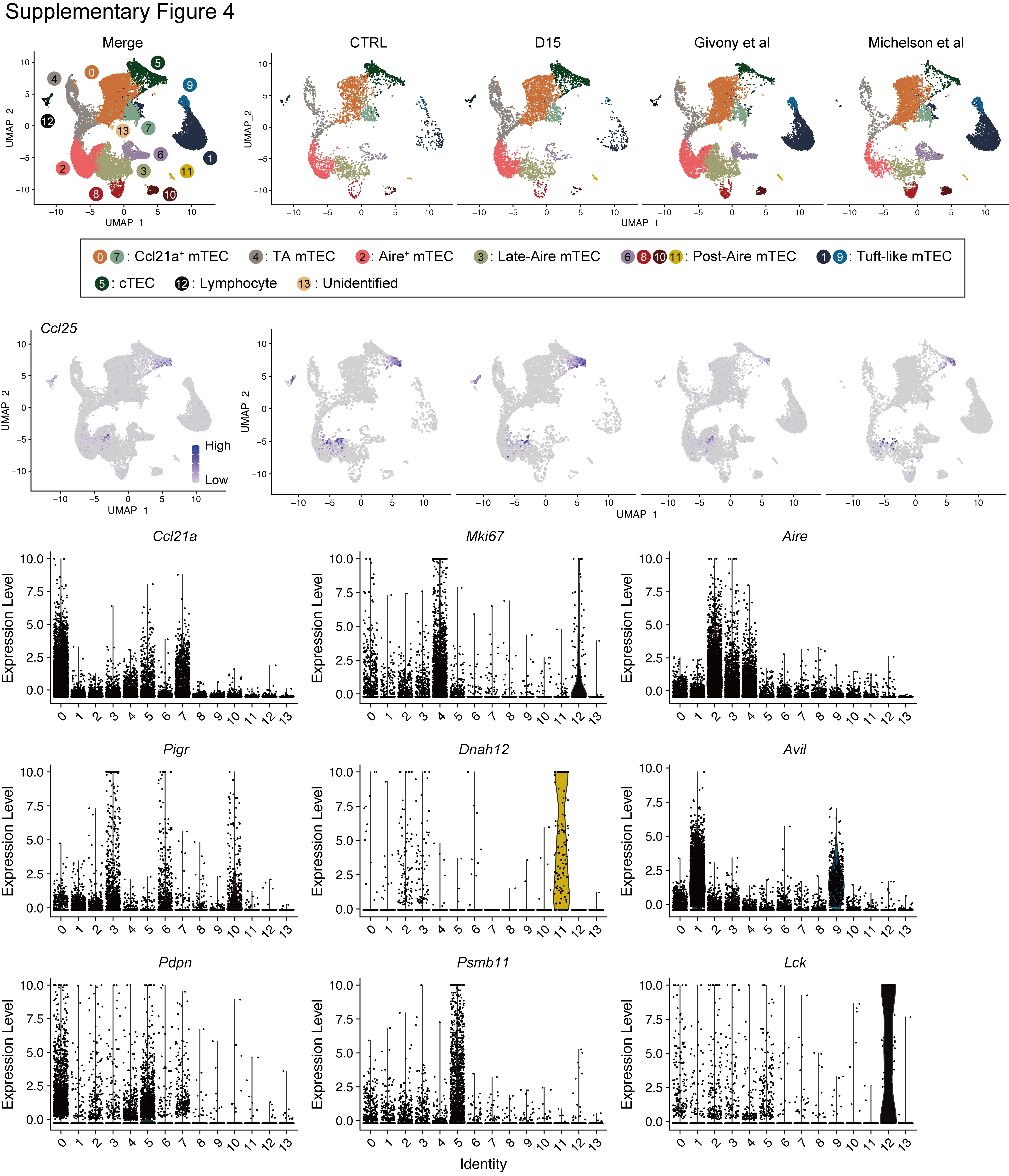

Supplement: Supplementary Figure 4 — Integrative analysis of scRNA-seq data. Our scRNA-seq data (CTRL and D15) are integrated with previously reported scRNA-seq data sets. (A) UMAP projection of integrated data, assignment of clusters, and expression of CCL25 in cell clusters. (B) Typical expression of marker genes in each cluster. [file Image_4.tif]
